# HER2 expression in urothelial carcinoma, a systematic literature review

**DOI:** 10.3389/fonc.2022.1011885

**Published:** 2022-10-21

**Authors:** Emilie Scherrer, Ashley Kang, Lisa M. Bloudek, Vadim S. Koshkin

**Affiliations:** ^1^ Seagen, Inc., Bothell, WA, United States; ^2^ Curta, Inc., Seattle, WA, United States; ^3^ Helen Diller Family Cancer Center, University of California San Francisco, San Francisco, CA, United States

**Keywords:** urothelial carcinoma, HER2, ErbB2, targeted therapy, systematic literature search

## Abstract

**Background:**

Urothelial carcinoma (UC) is a common malignancy with significant associated mortality. Recent clinical trials suggest an emerging role for HER2-targeted therapy. Testing for HER2 expression in UC is not part of current routine clinical practice. In consequence, the prevalence of HER2 expression in UC is not well defined.

**Methods:**

A systematic literature review (SLR) was conducted to characterize HER2 expression in both locally advanced unresectable or metastatic (LA/mUC) and earlier stage UC, classified as HER2+, HER2-low, HER2-. HER2+ was defined as an immunohistochemistry (IHC) score of 3+ or IHC 2+ and ISH/FISH+. HER2-low was defined as an IHC score of 2+ and ISH/FISH- or IHC 1+. HER2- was defined as an IHC score of 0. Weighted averages were calculated to generate an estimate of the population prevalence.

**Results:**

A total of 88 studies were identified, with 45, 30, and 13 studies investigating LA/mUC, earlier stage UC, and mixed stage/unspecified, respectively. The most common assays used were Dako HercepTest and Ventana Pathway anti-HER2/neu (4B5) for IHC to assess HER2 protein expression; Abbott PathVysion HER-2 DNA Probe Kit, FoundationOne CDx, and Guardant360 CDx for assessing HER2 gene amplification. The most frequently cited scoring guidelines were ASCO/CAP guidelines for breast cancer and gastric cancer, though most studies defined their own criteria for HER2 expression. Using the pre-specified definition, HER2+ prevalence ranged from 6.7% to 37.5% with a weighted average of 13.0% in LA/mUC. Only 1 study presented data that could be classified as HER2+ based on pre-specified criteria in earlier stage UC patients, and this study represented a likely outlier, at 76.0%.

**Conclusion:**

The results from this SLR help to shed light on HER2 expression in UC, a potentially clinically relevant biomarker-driven subpopulation for emerging HER2-directed regimens. Results of this SLR illuminate the variability in how HER2+ status expression levels are being assessed and how HER2+ is defined. Consensus on standardized HER2 testing and scoring criteria is paramount to better understand the clinical relevance in patients with UC.

## Introduction

Bladder cancer is a significant cause of mortality and morbidity globally, with 573,278 new cases and 212,536 deaths expected in 2020 (1). Histologically, approximately 90% of bladder tumors present as urothelial carcinoma (UC), and more than 90% of UC is located in the bladder (2). While the overall 5-year survival rate for all patients with UC is relatively high, at 68.6% in Europe and 77.1% in the US, the prognosis is worse for patients who have muscle-invasive disease at diagnosis (5-year survival rate of 37.5% in the US) or have distant metastases (5-year survival rate of 6.4% in the US) (3, 4).

Standard of care (SOC) for muscle-invasive UC is neoadjuvant cisplatin-based chemotherapy followed by radical cystectomy with pelvic lymph node dissection (4, 5). An estimated 25% of patients with muscle-invasive disease develop metastatic disease (6). For patients with locally advanced unresectable or metastatic UC (LA/mUC), SOC is systemic therapy with a platinum-based regimen for eligible patients, followed by switch-maintenance avelumab immunotherapy for patients who experience clinical benefit (7–9). Despite high initial response rates, most LA/mUC patients who start a platinum-based regimen will eventually experience disease progression and will relapse.

In recent years, several checkpoint inhibitors have gained US Food and Drug Administration (FDA) approval and are recommended for the treatment of LA/mUC patients in the second-line setting following platinum-containing chemotherapy, in the first-line setting for cisplatin-ineligible patients who have high programmed death-ligand 1 (PD-L1) expression or are ineligible for any platinum-based therapy, and as first-line maintenance therapy (7, 10–13). However, only a minority of patients with LA/mUC who receive immunotherapy will derive a benefit. Objective response rates (ORR) for patients who receive anti-programmed death receptor-1 (PD-1)/L1 therapy remain low in both the first-line setting (23% to 29%), and in the second-line setting and beyond (15% to 20%) (14–16). More recently, enfortumab vedotin-ejfv, an antibody-drug conjugate (ADC), received FDA approval for the treatment of LA/mUC patients who have previously received a PD-1 or PD-L1 inhibitor and platinum-containing chemotherapy or are ineligible for cisplatin-containing chemotherapy and have previously received one or more prior lines of therapy (17). Other recent advances in this disease space include the accelerated approval of sacituzumab govitecan-hziy for patients progressing on platinum-based chemotherapy and an immune checkpoint inhibitor and approval of erdafitinib in patients whose tumors harbor FGFR3 alterations following progression on platinum-containing therapy (18, 19).

The low response rates with current treatment options in LA/mUC indicate that there remains a large unmet need for effective treatment options. Among potential therapeutic targets is the human epidermal growth factor receptor 2 (*HER2*) gene (also referred to as *ERBB2*), which plays a role in regulating cell growth, differentiation, and survival. While HER2 is known to be overexpressed in various tumors, there are currently conflicting data on whether HER2 overexpression is an oncogenic driver and whether it is a prognostic marker in UC, as it has been demonstrated in breast and gastric cancer (20–26). Targeting HER2 has led to substantial survival gains in HER2+ breast and gastric cancer. In metastatic breast cancer, the treatment landscape drastically changed with the introduction of HER2-targeted therapies, which have been successful in extending overall survival in patients with HER2-expressing tumors (27–32). Similarly, in gastric cancer, the ToGA trial critically demonstrated the efficacy of HER2 agents for patients with HER2+ advanced gastric and gastroesophageal junction cancer, and HER2-targeted agents are now considered standard of care for these patients (32). As such, HER2 testing has become a clinical routine and guidelines for HER2 testing have been established for breast and gastric cancer.

While the therapeutic value of HER2-targeted therapies has been demonstrated in breast and gastric cancer, conventional anti-HER2 therapies such as trastuzumab and tyrosine kinase inhibitors (apatinib, neratinib, and lapatinib) have failed to improve outcomes in UC (33). Multiple novel treatments have been or are currently being developed to target patients with HER2+ UC, such as ADCs (33, 34). For example, disitamab vedotin (DV), also known as RC48-ADC, a HER2-targeted ADC conjugated to the microtubule disrupting agent monomethyl auristatin E (MMAE) *via* a protease cleavable linker, recently received an FDA breakthrough designation for previously-treated, platinum-eligible patients with HER2 expressing advanced UC, and has demonstrated encouraging results (35, 36).

Testing for HER2 expression in UC is not part of current routine practice. Estimates of the proportion of UC patients who are HER2+—and subsequently may benefit from these targeted therapies—are uncertain. Available literature on this topic cites wide ranges for the proportion of patients with UC whose tumors express HER2 (37). Furthermore, there are no standardized UC criteria to assess HER2 status, and guidelines for breast cancer, gastric cancer, or other criteria are often relied on (38, 39). As such, a comprehensive review of existing literature is needed to determine the prevalence of HER2 expression in patients with UC to better understand the potential role that emerging HER2 directed regimens may play in this patient population. This SLR was conducted to determine (a) the prevalence of HER2 expression in UC, and specifically in both earlier stage UC and LA/mUC, classified as HER2+, HER2-low, or HER2-; (b) how HER2 expression and amplification were assessed; and (c) the concordance between results from different tests.

## Materials and methods

### SLR methods

The SLR was conducted in accordance with Preferred Reporting Items for Systematic Review and Meta-Analysis Protocols (40). Searches were focused on studies published from January 2000 to October 2021 in MEDLINE and Embase databases. Abstracts presented at the following conferences held between 2019 and 2021 were also searched: American Society of Clinical Oncology (ASCO), American Society of Clinical Oncology Genitourinary Cancers (ASCO-GU), European Society for Medical Oncology (ESMO), Society of Urologic Oncology (SUO), American Urological Association (AUA), and European Association of Urology (EAU).

Studies which reported HER2 protein expression or gene amplification data for UC tumor samples were included. Reviews, case reports, case series, letters, or editorials were excluded, as were non-English language studies. Clinical trials of HER2+ patients were included if HER2 expression was tested as part of enrollment criteria, and the proportion was reported. Studies which investigated cultured tumor cell lines or non-human samples were excluded.

Studies were screened for inclusion and exclusion criteria by two independent reviewers. Disagreements between the two reviewers were resolved through discussion, with a third reviewer making the final decision, if needed. Data were extracted by one reviewer and validated by an independent reviewer.

### Definitions

HER2+ was defined as an immunohistochemistry (IHC) score of 3+ or IHC 2+ and *in situ* hybridization ISH+/fluorescence *in situ* hybridization FISH+. For studies to contribute data for HER2+, both IHC and ISH/FISH had to be conducted. In some studies, HER2+ was either defined differently (e.g., HER2+ as IHC 3+ or 2+ without confirmation by FISH) or left undefined. These studies were included in the category HER2+ (all studies). HER2-low was defined as an IHC of 2+ and ISH/FISH- or IHC 1+. HER2- was defined as an IHC score of 0.

### Statistical analysis

Weighted averages were calculated for the proportion of patients who were HER2+, HER2-low, and HER2- to generate an estimate of the proportion of patients in each HER2 expression category. Weighted average calculations were considered the point estimate.

## Results

### Search results

**Figure 1:**
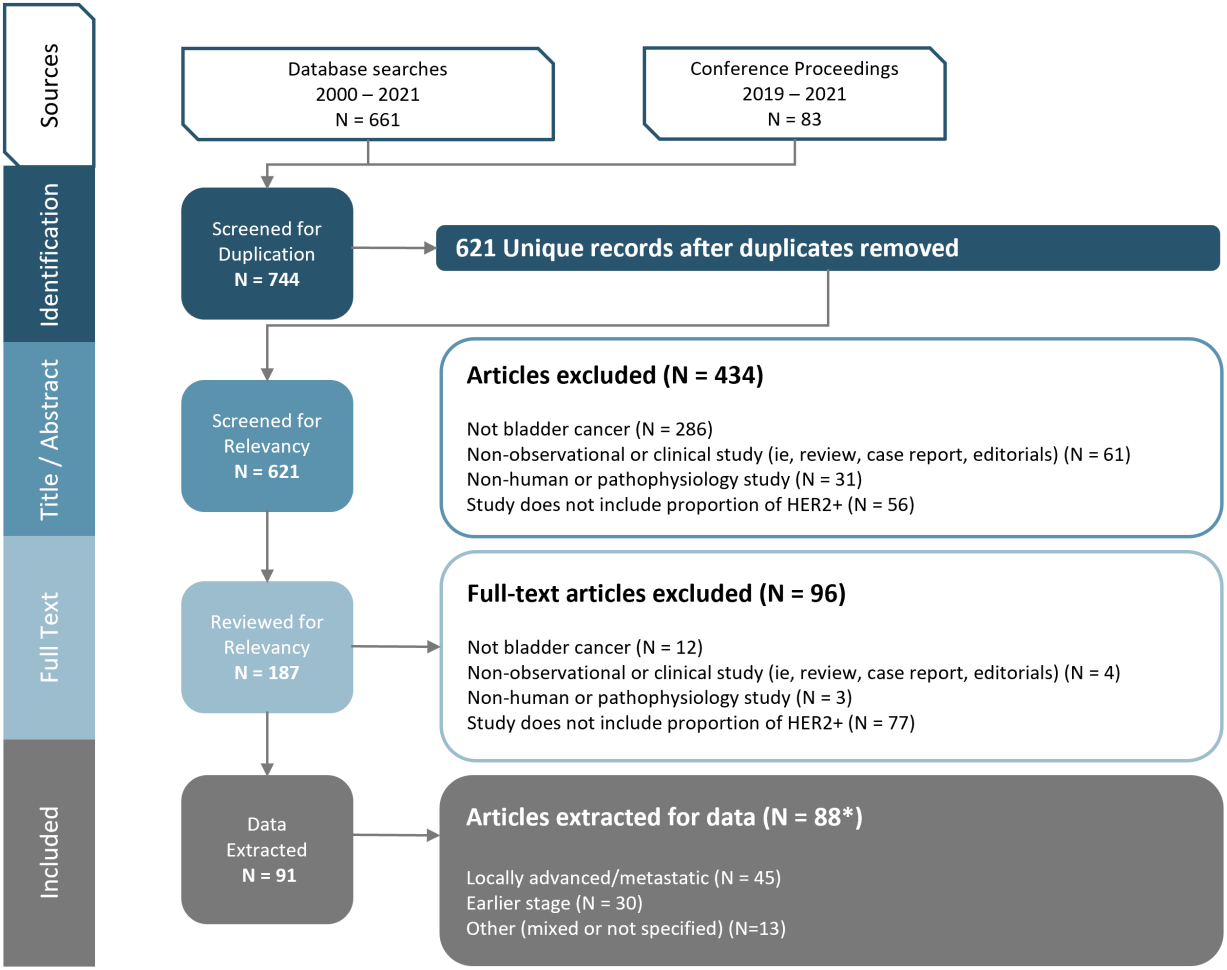
PRISMA Diagram. Figure footnote: *3 studies and 2 abstracts presented duplicate data, so data was extracted only once for each respective study.

Among 661 database references and 83 congress abstracts screened, 91 publications (representing 88 unique studies) were retained after full-text review (**Figure 1**). Of the 88 unique studies that reported data for HER2 status, 45 studies investigated LA/mUC patients, 30 studies investigated earlier stage UC (stage I-IIIA) patients, and 13 studies investigated a mixed earlier stage UC and LA/mUC patient population (n=7) or did not specify (n=6).

The majority of publications that met inclusion criteria were observational studies (75 of 88), with the remaining 13 comprised of clinical trials or pooled analyses of clinical trial enrollment screening.

### Assessment of HER2 expression and amplification

A total of 65 studies specified the type of test, assay, or antibody used. The most common IHC tests used were Dako HercepTest (IHC; n=18) and Ventana Pathway anti-Her2/neu (4B5) (IHC; n=6), and gene amplification tests were Abbott PathVysion HER-2 DNA Probe Kit (ISH; n=12), FoundationOne CDx (NGS; n=4), Guardant360 CDx (NGS; n=3), and Illumina NextSeq/MiSeq/HiSeq (NGS; n=3). A total of 41 studies used at least one assay other than the ones listed above, and 23 studies did not specify what test or assay was used. Assay counts were not mutually exclusive as some studies used multiple assays.

Various criteria were used to define HER2 expression, and most studies defined their own criteria (n=46) or did not mention criteria in the study methods (n=28). The most frequently cited scoring guidelines were ASCO/CAP guidelines for breast cancer (2007, 2013, and/or 2018) (n=13) and gastric cancer (n=5). Two studies cited ASCO/CAP guidelines but did not specify which guideline year was used. Only 1 study explicitly defined *ERBB2* amplification (≥3.5 copies) (41). Study counts were not mutually exclusive as some studies used more than 1 criterion or guideline.

### HER2 expression

LA/mUC. Of the total 28 LA/mUC studies that conducted IHC, 11 presented data that could be categorized into HER2+, HER2-low, and HER2- using pre-defined criteria (22, 36, 41–49). HER2+ ranged from 6.7% to 37.5% with a weighted average of 13.0% (4 studies, n=862) (**Table 1**) (22, 36, 42, 43). HER2-low ranged from 13.4% to 56.3% with a weighted average of 17.5% (2 studies, n=166) (**Table 2**) (22, 43). HER2- ranged from 0% to 79.9% with a weighted average of 39.5% (9 studies, n=803) (**Table 3**) (22, 41, 43–49).

**Table 1:** Overview of HER2+.

Study	Study design		N	HER2+	HER2+ criteria and assay	
LA/mUC						
Fleischmann 2011	Observational		150	6.7%	IHC 3+ or 2+ and FISH+; Dako HercepTest (IHC), Abbott/Vysis PathVysion HER2 DNA Probe Kit (FISH)	
Oudard 2015	Clinical trial, phase II		563	13.3%	IHC 3+ or 2+ and FISH+; Dako HercepTest (IHC), Dako HER2 FISH pharmDx Kit (FISH)	
Sheng 2021	Clinical trial, phase II		133	15.8%	IHC 3+ or 2+ and FISH+; Ventana PATHWAY anti-HER-2/neu (4B5) (IHC)	
Banerji 2019	Clinical trial, phase I		16	37.5%	ASCO/CAP guidelines for breast and gastric cancer; IHC 3+ or 2+ and ISH+; N/A	
**Weighted average of HER2+ defined as IHC 3+ or IHC 2+ and ISH/FISH+: 13.0%**						
Earlier stage UC						
Latif 2003		Observational	25	76.0%		IHC 3+ or 2+ and FISH+; Ventana monoclonal anti-human HER-2 protein CB11 (IHC), Vysis (FISH)
**Weighted average of HER2+ defined as IHC 3+ or IHC 2+ and ISH/FISH+: 76.0%**						

ASCO/CAP, American Society of Clinical Oncology/College of American Pathologists; FISH, fluorescence in situ hybridization; FISH+, FISH positive; HER2, human epidermal growth factor receptor; HER2+, HER2 positive; IHC, immunohistochemistry; N/A, not available.

**Table 2:** Overview of HER2-Low.

Study	Study design	N	IHC 1+	IHC 2+	ISH	HER2-Low	Assay
LA/mUC							
Fleischmann 2011	Observational	150	11.4%	4.7%	n=147FISH+: 8.8%Borderline +: 4.8%FISH-: 86.4%	13.4%	Dako HercepTest (IHC)Abbott/Vysis PathVysion HER2 DNA Probe Kit (FISH)
Banerji 2019	Clinical trial, phase I	16	12.5%	56.3%	Performed only for 2+ (n=16)ISH+: 11.1%/ISH-: 43.8%ISH unevaluable: 6.3%	56.3%	N/A
**Weighted average of HER2-Low defined as IHC 2+ and ISH/FISH- or IHC 1+: 17.5%**							
**Earlier stage UC**							
Latif 2003	Observational	25	4.0%	16.0%	FISH+: 8.0%	20.0%	Ventana monoclonal anti-human HER-2 protein CB11 (IHC), Vysis (FISH)
**Weighted average of HER2-Low defined as IHC 2+ and ISH/FISH- or IHC 1+: 20.0%**							

FISH, fluorescence in situ hybridization; FISH+, FISH positive; FISH-, FISH negative; HER2, human epidermal growth factor receptor; IHC, immunohistochemistry; ISH, in situ hybridization; ISH+, ISH positive; ISH-, ISH negative; N/A, not available.

**Table 3:** Overview of HER2-.

Study	Study design	N	IHC 0	ISH	HER2–	Assay
LA/mUC						
Banerji 2019	Clinical trial, phase I	16	0%	Performed only for 2+ (n=16)ISH+: 11.1%/ISH-: 43.8%ISH unevaluable: 6.3%	0%	N/A
Goodman 2016	Observational	11	0%		0%	Dako HER2 monoclonal mouse anti-human (IHC)
Carlsson 2015	Observational	72	11.1%		11.1%	Dako anti-HER2 rabbit polyclonal antibody A0485 (IHC)
Gårdmark 2005	Observational	86 (H)86 (T)90 (M)	H 33.7%T 52.3%M 15.6%		H 33.7%T 52.3%M 15.6%	Dako HercepTest (IHC)
Wülfing 2009	Clinical trial, phase II	57	22.8%		22.8%	Dako HercepTest (IHC)
Moktefi 2018	Observational	178	‘07: 32%‘13: 32%	n=51FISH+: 29.4%/FISH-: 39.2%	32%	Dako anti-HER2 rabbit polyclonal antibody A0485 (IHC)Dako HER2 FISH pharmDx Kit (FISH)
Cimpean 2017	Observational	34	47.1%		47.1%	Bond Oracle Human Epidermal Growth Factor Receptor 2 (IHC)
Choudhury 2016	Clinical trial, phase II	23	65.2%		65.2%	Dako HercepTest (IHC)
Fleischmann 2011	Observational	150	79.9%	n=147FISH+: 8.8%Borderline +: 4.8%FISH-: 86.4%	79.9%	Dako HercepTest (IHC)Vysis PathVysion HER2 DNA Probe Kit (FISH)
**Weighted average of HER2- defined as IHC 0: 39.5%**						
Earlier stage UC						
Latif 2003	Observational	25	20.0%		20.0%	Ventana monoclonal anti-human HER-2 protein CB11 (IHC), Vysis (FISH)
Tabriz 2021	Observational	84	23.8%		23.8%	Dako anti-HER2 rabbit polyclonal antibody A0485 (IHC)
Chakravarti 2005	Pooled analysis of clinical trials	55	40.0%		40.0%	Zymed (IHC)
Coogan 2004	Observational	54	40.7%		40.7%	Ventana monoclonal anti-human HER-2 protein CB11 (IHC)
Jimenez 2001	Observational	80	51.3%		51.3%	Dako c-erbB-2 primary antibody (IHC)
Bolenz 2010	Observational	198	73.9%		72.2%	Dako anti-HER2 rabbit polyclonal antibody A0485 (IHC)
**Weighted average of HER2- defined as IHC 0: 48.4%**						

FISH, fluorescence *in situ* hybridization; FISH+, FISH positive; FISH-, FISH negative; H, HercepTest; HER2, human epidermal growth factor receptor; HER2-, HER2 negative; IHC, immunohistochemistry; ISH, in situ hybridization; ISH+, ISH positive; ISH-, ISH negative; M, modified HercepTest; N/A, not available; T, target score.

Across all studies (35 studies) reporting HER2+ in LA/mUC patients with or without pre-determined criteria, HER2+ ranged from 4.3% to 83.3% with a weighted average of 25.4% (19 studies, n=2,268) (**Table 4**) (21, 22, 26, 36, 42, 45, 47, 50–58). *ERBB2* amplification ranged from 2.0% to 22.6% with a weighted average of 7.8% (16 studies, n=5,641) (**Table 5**) (41, 59–73).

**Table 4:** Overview of HER2+ across all studies.

Study	Study design		N	HER2+	HER2+ criteria and assay	
LA/mUC						
Bellmunt 2015	Observational		93 (Greece)88 (Spain)	4.3% (Greece)21.6% (Spain)	IHC 3+; N/A	
Fleischmann 2011	Observational		150	8.7%	IHC 2+ or 3+; Dako HercepTest (IHC), Abbott/Vysis PathVysion HER2 DNA Probe Kit (FISH)	
Grigg 2021	Observational		85	10.6%	2018 ASCO/CAP guidelines for breast cancer; Ventana PATHWAY anti-HER-2/neu (4B5) (IHC)	
Oudard 2015	Clinical trial, phase II		563	10.8%	IHC 2+ or 3+ and FISH+; Dako HercepTest (IHC), Dako HER2 FISH pharmDx Kit (FISH)	
Powles 2017	Clinical trial, phase III		446	13.5%	IHC 2+ or 3+; Novocastra antibodies HER2 (NCL-CBE-356) (IHC)	
Rink 2012	Observational		22	18.2%	IHC 3+; CTC Veridex CellSearch tumor phenotyping reagent HER2/neu (IHC)	
de Pinieux 2004	Observational		36	25.0%	++; Novocastra HER-2/neu antibody CB11 (IHC)	
Zhou 2021	Clinical trial, phase Ib/II		14	28.6%	IHC 3+, IHC 2+ and ISH+; N/A	
Sheng 2021	Clinical trial, phase II		133	32.3%	IHC 2+ or 3+; Ventana PATHWAY anti-HER-2/neu (4B5) (IHC)	
Banerji 2019	Clinical trial, phase I		16	37.5%	ASCO/CAP guidelines for breast and gastric cancer; IHC 3+ or ISH+; N/A	
Wülfing 2009	Clinical trial, phase II		57	43.9%	IHC 2+ or 3+; Dako HercepTest (IHC)	
Necchi 2015	Observational		52	46.2%	IHC 2+ or 3+; N/A	
Soria 2017	Observational		252	47.6%	IHC 2+ or 3+; Dako HercepTest (IHC)	
Hussain 2007	Clinical trial, phase II		109	52.3%	IHC 2+ or 3+; Dako HercepTest (IHC)	
Goodman 2016	Observational		11	54.5%	IHC 2+ or 3+; Dako HER2 monoclonal mouse anti-human (IHC)	
Kumar 2015	Observational		9	66.7%	IHC 2+ or 3+; Novocastra HER-2/neu monoclonal antibody clone CB11 (IHC)	
Gandour-Edwards 2002	Observational		39	71.8%	IHC 2+ or 3+; BioGenex c-erbB2 primary antibody (IHC)	
Wester 2002	Observational		21	81.0%	>67% of tumor cells should be stained; staining should be moderate to intense (++ or +++); staining pattern should be membranous, with or without concomitant cytoplasmic staining; Dako anti-HER2 rabbit polyclonal antibody A0485 (IHC)	
Carlsson 2015	Observational		72	83.3%	IHC 2+ or 3+; Dako anti-HER2 rabbit polyclonal antibody A0485 (IHC)	
**Weighted average: 25.4%**						
Earlier stage UC						
Laé 2010		Observational	1,005	11.4%		IHC 2+ or 3+; Dako anti-HER2 rabbit polyclonal antibody A0485 (IHC)
Kiss 2017*		Observational	127	18.9%		IHC 3+; Dako HercepTest (IHC)
Mejri 2014		Observational	21	19.0%		IHC 2+ or 3+; Leica antibody NCL-N-CD11 (IHC)
Eriksson 2017		Observational	292	21.1%		HER2 amplified tumors with IHC 2+ or 3+ in >10% of cells; Ventana PATHWAY anti-HER-2/neu (4B5) (IHC)
Naruse 2010		Observational	46	21.7%		IHC 3+; Dako HercepTest (IHC)
Kossai 2021*		Observational	32	25.0%		IHC 2+ or 3+; N/A
Coogan 2004		Observational	54	26.0%		IHC 2+ or 3+; Ventana monoclonal anti-human HER-2 protein CB11 (IHC)
Jimenez 2001		Observational	80	27.5%		IHC 2+ or 3+; Dako c-erbB-2 primary antibody (IHC)
Bolenz 2010		Observational	134	26.1%		IHC 1+, 2+, or 3+ (in ≥10% of tumor cells); Dako anti-HER2 rabbit polyclonal antibody A0485 (IHC)
Chiang 2019		Observational	41	29.3%		IHC 2+ or 3+; Ventana Benchmark (IHC)
Soria 2016		Observational	354	35.6%		IHC 2+ or 3+; Dako HercepTest (IHC)
Hansel 2008*		Observational	53	35.8%		IHC 2+ or 3+; Ventana PATHWAY anti-HER-2/neu (4B5) (IHC)
Matsubara 2008		Observational	40	42.5%		IHC 2+ or 3+; Dako HercepTest (IHC)
Leite 2021		Observational	25	44.0%		N/A; Biocare EP3 clone (IHC)
Røtterud 2005		Observational	19	47.4%		++ or +++; BioGenex StrAviGen MultiLink Kit (IHC)
Tabriz 2021*		Observational	84	52.4%		IHC 2+ or 3+; Dako anti-HER2 rabbit polyclonal antibody A0485 (IHC)
Kolla 2008		Observational	90	55.6%		IHC 2+ or 3+; BioGenex CB11 antibodies (IHC)
Chakravati 2005		Pooled analysis of clinical trials	55	60.0%		IHC 1+, 2+, or 3+; Zymed (IHC)
Latif 2003		Observational	25	76.0%		IHC 2+ or 3+; Ventana monoclonal anti-human HER-2 protein CB11 (IHC), Vysis (FISH)
**Weighted average: 24.6%**						

ASCO/CAP, American Society of Clinical Oncology/College of American Pathologists; FISH, fluorescence in situ hybridization; FISH+, FISH positive; HER2, human epidermal growth factor receptor; HER2+, HER2 positive; IHC, immunohistochemistry; N/A, not available. *Overexpression.

**Table 5:** Overview of ERBB2 amplification.

Study	Study design	N	*ERBB2* Amplification	Assay
LA/mUC				
Jacob 2021	Observational	49	2.0%	N/A
Cabel 2018	Pooled analysis of clinical trials	44	2.3%	N/A
Pobel 2021	Pooled analysis of clinical trials	182	2.7%	N/A
Ross 2014	Observational	35	2.9%	N/A
Alhalabi 2021	Clinical trial, phase I	41	4.9%	N/A
Vandekerkhove 2021	Observational	104	6.7%	N/A
Almassi 2019	Observational	131	6.9%	N/A
Madison 2020	Observational	3,753	7.4%	N/A
Agarwal 2018	Observational	369	8.1%	N/A
Ross 2016	Observational	295	8.5%	N/A
Sarid 2019	Observational	60	10.0%	N/A
Millis 2015	Observational	284	11.6%	N/A
Villamar 2019	Observational	214	12.8%	N/A
Choudhury 2016	Clinical trial, phase II	23	13.0%	ThermoFisher Scientific Ion AmpliSeq Library Kit and Comprehensive Cancer Panel
Vandekerkhove 2017	Observational	26	19.2%	N/A
Fina 2016	Observational	31	22.6%	N/A
**Weighted average: 7.8%**				
Earlier stage UC				
Almassi 2019	Observational	491	9.0%	N/A
**Weighted average: 9.0%**				

ERBB2, Erb-B2 receptor tyrosine kinase 2; N/A, not available.

Earlier stage UC. Of the 25 earlier stage UC studies that conducted IHC, 6 presented data that could be categorized into HER2+, HER2-low, and HER2- using pre-defined criteria (24, 74–78). Only 1 study (n=25) presented data that could be classified as HER2+ (60.0%) and HER2-low (20.0%) in earlier stage UC patients (**Tables 1**, **2**) (74). HER2- ranged from 20.0% to 73.9% with a weighted average of 48.4% (6 studies, n=432) (**Table 3**) (24, 74–78).

Across all studies reporting HER2+ (17 earlier stage UC and 2 mixed early stage and LA/mUC which reported data for the earlier stage subgroup), HER2+ in earlier stage UC ranged from 11.4% to 76.0% with a weighted average of 24.6% (19 studies, n=2,577) (**Table 1**) (24, 37, 74–90). Only 1 study reported *ERBB2* amplification in earlier stage UC at 9.0% (n=491) (**Table 4**) (91).

### Concordance between HER2 overexpression and amplification

No studies identified in this SLR reported concordance for HER2 overexpression (IHC 3+) and gene amplification by NGS or gene amplification by ISH/FISH and NGS. Three studies outside of this SLR reported the level of concordance between HER2 overexpression and gene amplification, ranging from 71% to 91% (49, 70, 92). In patients with LA/mUC, 1 study reported tumor samples assessed with both IHC and ISH showed that 91% of tumors with IHC 3+ had HER2 gene amplification by ISH (70). In studies of UC overall, concordance between HER2 IHC 3+ and gene amplification by FISH and HER2 IHC 3+ and gene amplification by brightfield double *in situ* hybridization (BDISH), were 73.3% and 71%, respectively (49, 92).

## Discussion

There is a growing body of literature that suggests the importance of the *HER2* gene as a potentially clinically relevant biomarker in UC (33, 93, 94). To the best of our knowledge, this is the first study to systematically characterize the prevalence of HER2 expression in UC, as classified as HER2+, HER2-low, or HER2-. For patients with LA/mUC, we found published literature suggesting HER2+ to be present in 13-25% of patients and HER2-low status in up to 20%. This SLR only identified 1 earlier stage UC study that had available data to categorize into the pre-defined categories, reporting HER2+ prevalence of 60%, which is within the range that has been previously cited in literature (27.8%-85.2%) (93). However, reported ranges for HER2+ (for all studies regardless of criteria) were extremely variable for both LA/mUC (4%-83%) and earlier stage UC patients (11%-76%). As such, based on the findings of this SLR, HER2 expression did not seem to differ significantly between early stage and more advanced disease. It is notable however, that a recent, single-institution NGS study seemed to suggest that HER2 amplification occurred more frequently in patients with muscle invasive and metastatic BC than in patients with non-muscle invasive BC (95).

Wide ranges for HER2+ (all studies) can be attributed to the lack of optimized staining process and standardized criteria, specific for UC. The estimate for HER2+ using pre-defined criteria was lower than the estimate when all studies with HER2+ data, regardless of criteria, were included. Many studies used less rigorous criteria, such as IHC 2+ not confirmed by ISH/FISH, when defining HER2+. Had these studies tested for and confirmed patients by ISH/FISH, some likely would fall into the HER2-low classification, rather than HER2+. Overall, this highlights the importance of generating more rigorous data for HER2+ status in patients with urothelial cancer and its clinical relevance, using more optimized and standardized assays and scoring criteria.

Differences in scoring over time also likely contributed to the variability observed, as the same patients can be scored differently based on the guideline used. In one study, nearly half of patients were reclassified from IHC score 1+ to 2+ based on the shift in definitions from the 2007 to 2013 ASCO/CAP guidelines. The substantial shift in IHC scores from 1+ to 2+ directly affected the number of cases that were eligible for FISH testing, as 58 more patients—among a total of 98 patients—were FISH tested (48). Consequently, the proportion of patients who were HER2+ (IHC 3+ or IHC 2+/FISH+) increased from 15.3% using the 2007 guidelines to 28.6% using the 2013 guidelines.

There is a great need for optimization and standardization in testing methodology as well as algorithms for test result interpretation in UC. Published studies in breast and gastric cancer demonstrate the significance in validating and developing standardized testing in order to accurately select patients who may benefit from HER2-targeted therapies (96–98). While characteristic expression and HER2 scoring systems have been established in breast and gastric cancer, they have not in UC (99). It has been suggested that the pattern of HER2 staining on tumor cells in UC does not exactly mirror either breast or gastric staining patterns, but rather is a mix of the two—circular and patchy (100). Moreover, when assessing UC samples, FISH testing is rarely performed in routine clinical practice, unlike that for breast and gastric tumors. Additionally, it is important that studies assess factors contributing to variability in HER2 testing in order to reduce variation and improve validity (96). One study identified various factors, such as tumor location and Lauren classification, that affect HER2 testing results in gastric cancer; however, similar data in UC is currently unavailable (96). Overall, more comprehensive guidelines for HER2 testing in UC should be developed.

Very few studies identified in this SLR provided information on concordance between test results. In general, there is a dearth in published literature on concordance between detection methods in UC, with some studies focusing on reporting concordance between primary tumors and metastases (37, 79, 101). This SLR yielded 3 studies that reported concordance rates between differing techniques of assessing HER2 status, ranging from 71% to 91% (49, 70, 92). Overall, published literature seems to suggest that IHC 3+ and ISH/FISH produce similar results in UC. Future studies should aim to investigate concordance for HER2 overexpression and gene amplification by NGS, as adoption of NGS in clinical practice has increased in recent years.

A caveat of this SLR is the limited availability of IHC data. Though the presented definitions of HER2+, HER2-low, and HER2- are consistent with other tumor types, they require both IHC and ISH/FISH testing, which often limits the number of studies that could contribute data. ISH data were also often not presented, limiting the number of studies which contributed data to the estimates of HER2+ and HER2-low. These findings highlight the inconsistency in testing methods and assays currently used for HER2 testing in UC.

Differences in patient population, including staging and treatment history, as well as the timeframe and location of the included studies, may have also contributed to the wide ranges observed. For instance, location of primary tumor may affect HER2 overexpression. Patients with upper tract UC are more likely to have higher expression of HER2, with some studies reporting HER2 expression as an independent prognostic factor for patients with upper tract UC (102, 103). Heterogeneity of molecular subtypes may play a role as well. Luminal subtypes of bladder cancer are also characterized by overexpression of HER2 and are overrepresented in upper tract UC, which may help to explain higher expression rates in upper tract UC (104, 105). Additionally, geographic location and race/ethnicity of patients can affect HER2 status. One study found large differences in HER2 overexpression and/or amplification between Spanish and Greek patients and demonstrated that HER2 status varies between populations, suggesting etiologic heterogeneity (21). Lastly, an important difference to note is that some patients are more likely to get HER2 testing and more extensive path review of their tumors due to factors such as where patients receive care (e.g., academic institutions or community-based), geographic region, and insurance status.

Weighted averages were calculated in order to generate a summary estimate but do not account for heterogeneity across studies or statistical uncertainty in the pooled estimates.

Lastly, different studies used different tissue to assess HER2 status for LA/mUC, and some studies may have used primary tissue of the metastatic tissue to assess HER2 status even when results were reported as LA/mUC.

## Conclusion

This review of published literature of HER2 expression in patients with LA/mUC revealed a wide reported range of prevalence, 6.7% to 37.5% for HER2+ and 13.4% to 56.3% for HER2-low. This variation may be attributed to heterogenous study populations, tissue samples collected, and testing methodologies. Results of this SLR help shed light on HER2 expression in UC, a potentially clinically relevant biomarker-driven population for emerging HER2-directed regimens, suggesting that up to half of all patients with UC may have some level of HER2 expression that could potentially be targeted. As these novel HER2-directed agents spark new interest in further understanding the role of HER2 expression, gene amplification, and mutations in UC, it is important to utilize well-characterized, optimized, and standardized assays as well as scoring, interpretation criteria, and data analysis for IHC, ISH/FISH, or NGS assays.

## Data availability statement

The original contributions presented in the study are included in the article/supplementary material. Further inquiries can be directed to the corresponding author.

## Author contributions

ES made key contributions to the study design, helped draft the manuscript, and revised the submitted article for important intellectual content. AK made key contributions to the study design, performed analysis, assisted with interpretation of results, and helped draft the manuscript. LB made key contributions to the study design, assisted with interpretation of results, and helped draft the manuscript. VK helped draft the manuscript and revised the submitted article for important intellectual content. All authors read and approved the final manuscript. They are accountable for all aspects of the work and in ensuring that questions related to the accuracy or integrity of any part of the work are appropriately investigated and resolved.

## Funding

This study was sponsored by Seagen Inc., Bothell, WA.

## Conflict of interest

AK and LB are employees of Curta, Inc., which received funding from Seagen Inc. in connection with this research. ES is an employee of Seagen Inc. and hold stock/stock options in Seagen Inc. VK has served in a consulting or advisory role for AstraZeneca, Clovis, Janssen, Pfizer, EMD Serono, Seagen, Astellas, Dendreon, Guidepoint, GLG and ExpertConnect; has received research funding for the institution from Endocyte, Nektar, Clovis, Janssen and Taiho.

## Publisher’s note

All claims expressed in this article are solely those of the authors and do not necessarily represent those of their affiliated organizations, or those of the publisher, the editors and the reviewers. Any product that may be evaluated in this article, or claim that may be made by its manufacturer, is not guaranteed or endorsed by the publisher.
